# Supplementary figures and images for: Fine grained compositional analysis of Port Everglades Inlet microbiome using high throughput DNA sequencing
Source: PeerJ. 2018 May 8;6:e4671. doi: 10.7717/peerj.4671 (PMC5947159; doi:10.7717/peerj.4671)

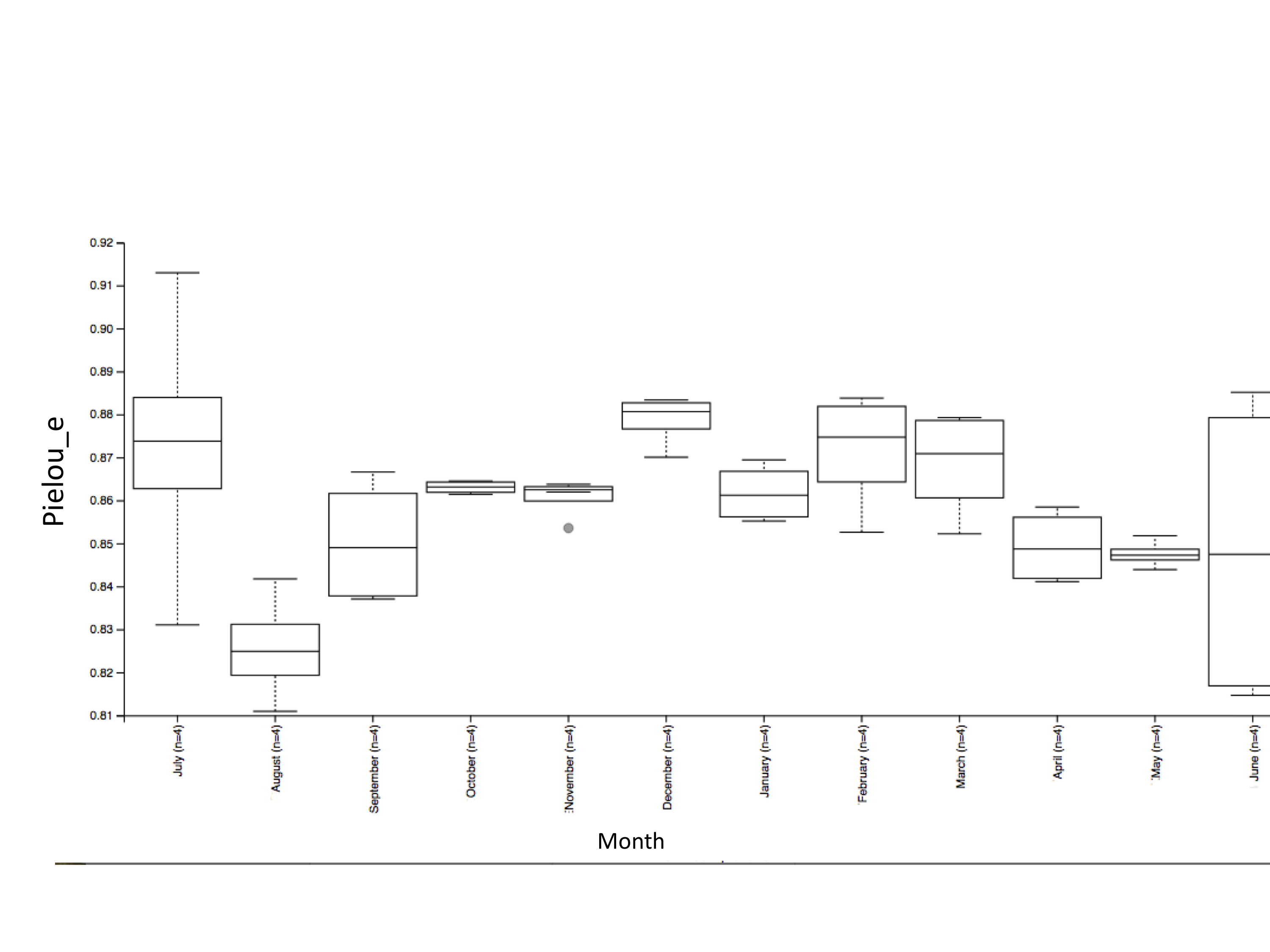

Supplement: Figure S1 — Boxplot depicting species evenness of samples during every month. There were significant differences in species evenness across all months (Kruskal–Wallis using Pielou’s evenness index H = 23.57 p = 0.015). The month of August had the lowest measure of species evenness. The month of December had the highest measure of species evenness. [file peerj-06-4671-s005.png]

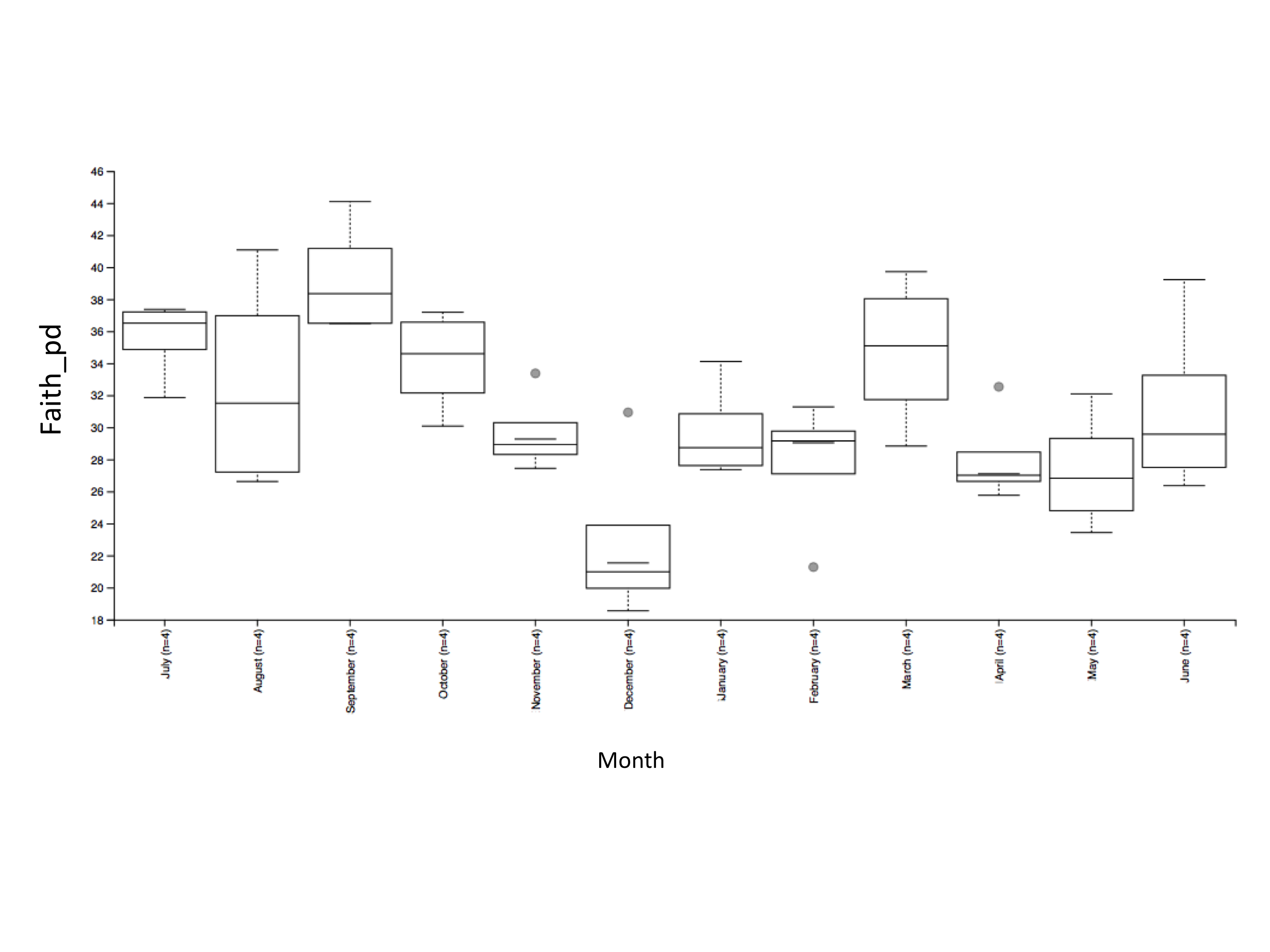

Supplement: Figure S2 — Boxplot depicting species richness of samples taken during every month. There were significant differences in species richness between different months (Kruskal–Wallis using Faith’s Phylogenetic Distance H = 24.57 p = 0.011). The month of December had the lowest measure of species richness while September had the highest measure of species richness. [file peerj-06-4671-s006.png]

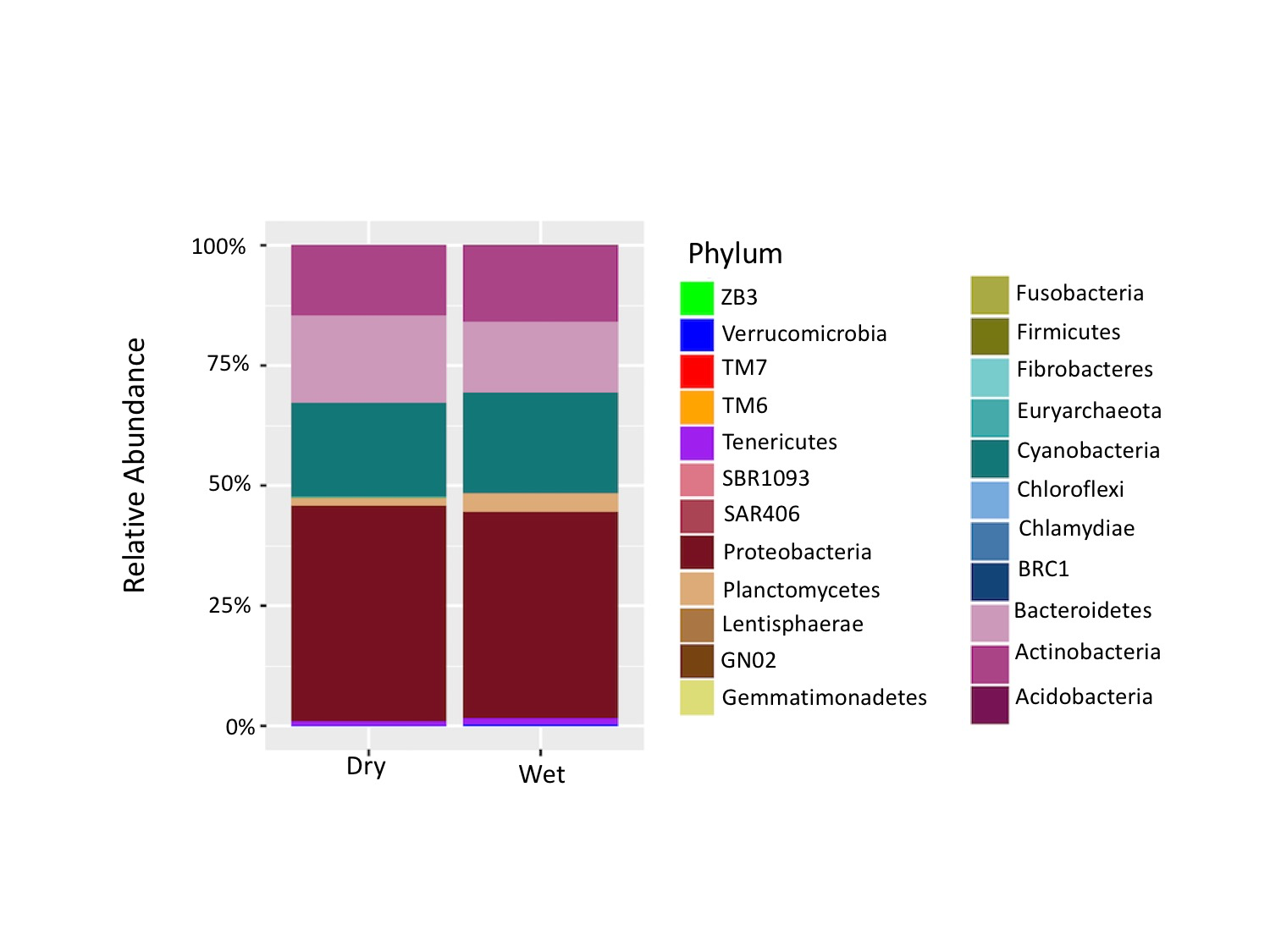

Supplement: Figure S3 — Stacked bar chart displaying seasonal fluctuations of relative abundance levels at Phylum-level classification. There were no statistical differences in relative abundances at phylum level throughout the year. [file peerj-06-4671-s007.png]

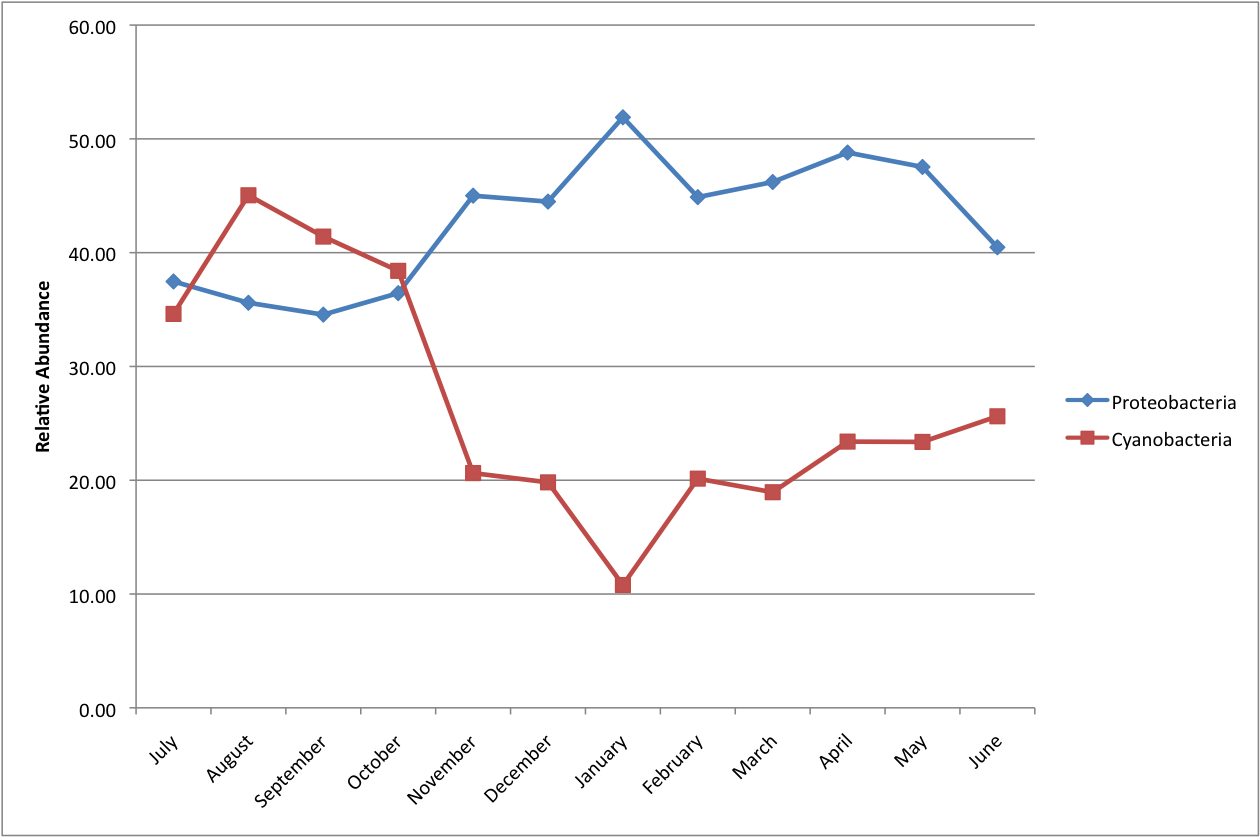

Supplement: Figure S4 — Interesting inverse trend observed in relative abundances between Proteobacteria and Cyanobacteria. [file peerj-06-4671-s008.png]
